# A New Fashion of Sleeping

**Published:** 1881-07

**Authors:** 


### A NEW FASHION OF SLEEPING.

The Chicago *Tribune* lately contained a lengthy article on the new fashion which is rapidly coming into vogue of husbands and wives occupying separate beds. The practice is said to be an European one, and is being received in New York with great favor. It has long been held by physicians that the habit of two persons occupying the same sleeping couch was a vicious one, as the superior magnetism of one would draw the vitality out of the other; and while one would awake in the morning refreshed with new energy, the other would be enervated and listless and hardly able to drag the body around during the day. Acting upon this European theory it is supposed that the dealers in beds and bedding have secretly organized a boom in their business, and knowing how a fashion will be followed by a people they are preparing for a season of prosperity. But, be this as it may, there is little doubt that the fashion of sleeping singly has its good as well as bad points, and it will be hailed with delight by those husbands whose wives are troubled with charity feet. It will also come as a sweet boon to the tired wife whose husband is a beautiful dreamer and habitually kicks her out of bed as he yells "I'll sell two thousand March at six seven-eighths." But on the rich old husband, whose young wife is kept awake by his asthma, and who will avail herself of the new fashion for a night of undisturbed repose, it will fall with a leaden hand, and his supply of magnetism being cut off he will droop and die and leave his cash so much the sooner. There are, as we said, many good features which will recommend themselves to different people upon reflection. Acting upon the new fashion it is said that landlords are already providing the bridal chambers in their hotels with two beds, thus aiming to leave nothing undone that will be conducive to the comfort of guests. Upon the whole the new fashion is one possessed of great merit.—*Peck's Sun.*

